# Optimizing healthcare for blood donors at risk of transfusion-transmissible infections: pre-implementation mixed methods protocol

**DOI:** 10.1080/16549716.2025.2540685

**Published:** 2025-08-12

**Authors:** Juan Macalupu, Elsa González-Lagos, Sarah Gimbel, Eduardo Gotuzzo

**Affiliations:** a Instituto de Medicina Tropical Alexander von Humboldt, Universidad Peruana Cayetano Heredia, Lima, Peru; b Department of Global Health, University of Washington Schools of Medicine and Public Health, Seattle, USA; c Department of Child, Family and Population Health Nursing, University of Washington School of Nursing, Seattle, USA

**Keywords:** Blood safety, infection control, systems analysis, digital health, stakeholder participation

## Abstract

In the Peruvian Amazon, a significant proportion of replacement blood donors test reactive for HIV, HTLV-1/2, and other transfusion-transmissible infections but often receive no subsequent care. Optimizing healthcare for these individuals can mitigate adverse outcomes by enabling early diagnosis and management. Effective coordination between Blood Bank and Infectious Diseases services is essential to provide comprehensive care. To address this challenge, this study applies an Implementation Science approach to design and assess an innovation that enhances healthcare for blood donors affected by transfusion-transmissible infections. A pre-implementation mixed methods study will be conducted at two hospitals in the Peruvian Amazon, employing the Consolidated Framework for Implementation Research (CFIR) and a systems engineering tool (mapping). The study will focus on the inner setting domain of CFIR and map the care continuum to identify contextual determinants that may influence implementation. It has three specific aims planned for the future: first, to use a convergent mixed methods approach to examine the care continuum of blood donors and the inner settings; second, to co-design an innovation through qualitative and participatory methods, integrating Blood Bank and Infectious Diseases services via the hospital information management system; and third, to evaluate the feasibility, appropriateness, and acceptability of the co-designed innovation through a pilot. Replacement blood donation and fragmented healthcare systems are common challenges in other low- and middle-income countries. The findings of this study can inform future implementation research and policy in similar settings.

## Paper context

- **Main findings**: This study will co-design and pilot an innovation to improve how blood donors at risk of transfusion-transmissible infections receive screening results, confirmatory testing, and care, by integrating Blood Bank and Infectious Diseases services through digital tools.
- **Added knowledge**: This study employs a participatory, mixed-methods implementation science approach to systematically assess and address gaps in blood donor healthcare, providing a scalable methodology while ensuring context-specific, actionable interventions for resource-limited settings.
- **Global health impact for policy and action**: This study will drive digital innovations in blood donor healthcare to strengthen blood safety and infectious disease control, while framing a pragmatic, adaptable approach for integrating fragmented healthcare systems in resource-limited settings.

## Background

Early diagnosis and management of blood donors potentially affected by transfusion-transmissible infections are opportunities that can be achieved through systematic screening in blood banks [1]. In Peru, blood banks screen for Human immunodeficiency virus (HIV), Human T-lymphotropic virus 1 and 2 (HTLV-1/2), Syphilis, Hepatitis B and C, and Chagas disease through methods that vary according to capacities [2]. In Peru and other low-, middle-, and upper-middle-income countries, screening for transfusion-transmissible infections is carried out to ensure transfusion safety, but blood donors are rarely informed of their reactive results, properly diagnosed, and managed when necessary [2–4]. An innovation that integrates Blood Bank and Infectious Diseases services [5], designed using an Implementation Science approach, may be feasible, appropriate and acceptable to optimise healthcare for at-risk blood donors.

In contrast to the United States of America, where almost all blood donations come from volunteers, about 90% of blood donations in Peru come from replacement donors (i.e. individuals who donate blood to replace the units used by a specific patient) [6]. Compared with voluntary donors, replacement donors show higher prevalence of transfusion-transmissible infections [7]. Providing optimal person-centred care to blood donors with reactive screening results in underserved settings can prevent further transmission and significantly reduce long-term individual complications, such as those associated with delayed initiation of antiretroviral therapy [8].

The population of the Peruvian Amazon lives with notable inequalities and gaps in health services [9]. In particular, indigenous communities are difficult to reach due to transportation, language, and cultural barriers, and therefore suffer from higher disparities [10,11]. Blood donors from the Amazon region test reactive for HIV, HTLV-1/2 and other transfusion-transmissible infections at higher rates than in other regions [2]. In Madre de Dios, the proportion of blood donors testing reactive for HIV and HTLV-1/2 is almost five and eight times, respectively, the national figure [2]. Madre de Dios and Ucayali have the highest incidence of Acquired immunodeficiency syndrome (AIDS) and HIV, respectively, in Peru [12]. Ucayali is also home to the largest number of Shipibo-Konibo communities (102/153), where HTLV-1/2 is endemic and can be devastating [13–15].

Optimal healthcare for blood donors should include counseling, diagnosis, and management of transfusion-transmissible infections, tailored to the specific characteristics of a region [16]. However, donor notification of reactive transfusion-transmissible infections screening and confirmation of possible infection are processes that are neither mandatory nor regulated in Peru [12]. This gap is not only impractical, but also an ethical dilemma, leaving individuals uninformed and at risk of preventable complications [17]. In 2023, 24361 blood donors in Peru had a reactive screening result, but no data were published on the proportion of confirmatory testing or notification [18].

Effective coordination between Blood Bank and Infectious Diseases services has the potential to improve healthcare for populations at risk of having transfusion-transmissible infections [19,20]. Existing information management systems such as SISGALENPLUS in Peru, a software designed to link hospital services of the Ministry of Health [21], could be used to coordinate these processes [5]. However, this system was not specifically designed for the problem or settings of interest, which could affect its feasibility, appropriateness and acceptability [22].

Using SISGALENPLUS, a tailored innovation could efficiently address this practical problem. To increase the likelihood of successful implementation, an in-depth study is needed to identify contextual determinants before designing the innovation [23]. It is also recommended to study implementation determinants before implementing an innovation [23]. Therefore, this study is guided by the Consolidated Framework for Implementation Science (CFIR) as a determinant framework [23,24].

In this pre-implementation study, we aim to design and test an innovation, which optimizes the healthcare for blood donors at risk of transfusion-transmissible infections in Madre de Dios and Ucayali. We will gather holistic information on contextual determinants before co-designing the innovation, by studying the inner setting domain and mapping current bottlenecks in the blood donor care continuum. The co-design of the innovation will be participatory, based on a human-centred design approach. Finally, we will assess the feasibility, appropriateness and acceptability of the innovation as antecedent implementation determinants. As the practical problem addressed in this project is common in other low- and middle-income countries, this protocol will inform future implementation research.

## Objectives

1. To design and pilot-assess a feasible, appropriate and acceptable innovation aimed to optimise the healthcare delivery system for blood donors potentially affected by transfusion-transmissible infections in two tertiary hospitals from the Peruvian Amazon

- Specific aim 1: To identify areas for improvement in the healthcare delivery of blood donors potentially affected by transfusion-transmissible infections by in-depth describing the care continuum of blood donors and the inner settings of Blood Bank and Infectious Diseases services
- Specific aim 2: To co-design an innovation to address identified areas for improvement, based on the care continuum of blood donors and the inner settings of Blood Bank and Infectious Diseases services
- Specific aim 3: To pilot and assess the feasibility, appropriateness and acceptability of the co-designed innovation according to healthcare workers of Blood Bank and Infectious Diseases services and blood donors

## Methods

### Preliminary work

Between October 2023 and June 2024, we conducted an informal situational analysis through in-person and remote discussions with local investigators from both Amazon regions [25]. Local investigators have provided us with preliminary information about local resources and the processes of transfusion-transmissible infections (TTI) screening, diagnosis and management, as well as their perspectives on the problem. We have refined the study protocol after this input. The following activities have not been implemented at the moment of submission.

### Design

#### Specific aim 1

The design of the first phase is convergent mixed methods with equal priority: Quantitative (Quan) + Qualitative (Qual) (Graphic 1) [26]. We will gather comprehensive information related to the care continuum of blood donors and the inner settings of both ‘Santa Rosa’ and ‘Amazónico’ hospitals. Results from both strands will be gathered and analyzed separately, then integrated and presented as narrative discussion and joint display.

**Figure:**
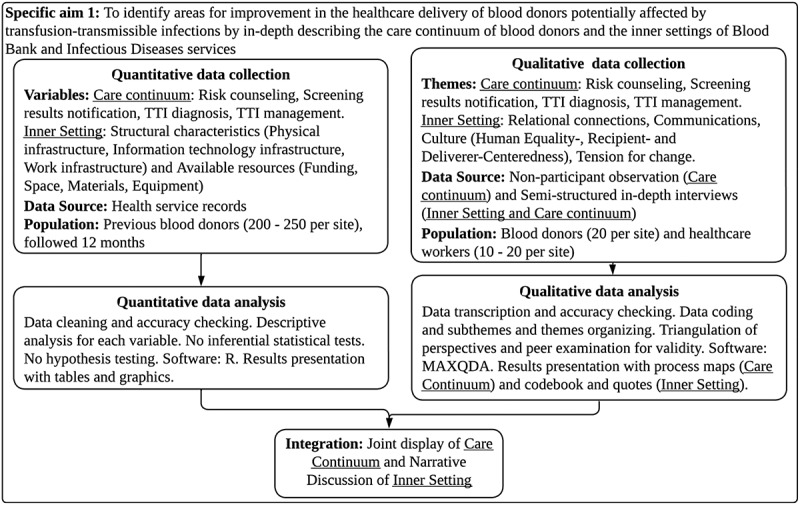
Graphic 1: Convergent mixed methods design for specific aim 1.

The quantitative strand will gather secondary data regarding the care continuum (risk counseling, screening results notification, TTI diagnosis and TTI management) of previous blood donors with reactive screening results from blood donation to 12 months after. This strand will also gather secondary data regarding structural characteristics (physical infrastructure, information technology infrastructure, work infrastructure) and available resources (funding, space, materials, equipment) of Blood Bank and Infectious Diseases services. After accuracy checking and cleaning, quantitative data will be described using R software.

The qualitative strand will gather primary data regarding the care continuum (risk counseling, screening results notification, TTI diagnosis and TTI management) of blood donors through non-participant observation for process mapping and semi-structured in-depth interviews to blood donors and healthcare workers. This strand will also gather primary data regarding relational connections, communications, culture and tension for change through semi-structured in-depth interviews to healthcare workers of Blood Bank and Infectious Diseases services. After verbatim transcription and accuracy checking, qualitative data will be coded and analyzed thematically using MAXQDA software.

#### Specific aim 2

The design of the second phase is qualitative, participatory and formative, in which the innovation will be co-designed (Graphic 2). The innovation will work on the SISGALENPLUS software as the tool for integrating Blood Bank and Infectious Diseases services. This phase consists of four successive stages: Stakeholder analysis and results dissemination, Innovation co-design, Stakeholders feedback, and Innovation refinement.

**Figure:**
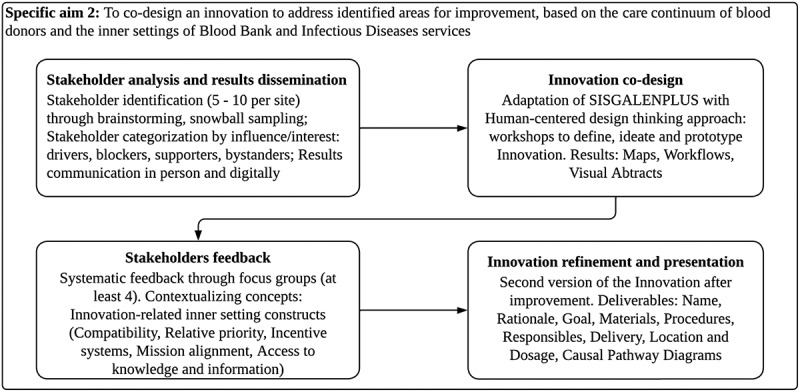
Graphic 2: Qualitative, formative and participatory design for specific aim 2.

Stakeholder analysis and results dissemination: Local investigators will systematically analyze regional stakeholders [27]: Stakeholder identification through brainstorming and snowball sampling; stakeholder categorization by consensus: influence/interest maps (drivers, blockers, supporters, bystanders). Stakeholders will be contacted by mail, letter and phone call. Identified regional stakeholders and others identified by themselves will be invited to share results from specific aim 1 with them in person. Those who cannot participate will be invited for a virtual meeting. Results will also be provided by email to all stakeholders.

Innovation co-design: We will co-design a first version of an Innovation to adapt SISGALENPLUS, based on the areas of improvement identified from aim 1 [28,29]. Local investigators will be responsible for developing the first version of the Innovation for both ‘Santa Rosa’ and ‘Amazónico’ Hospitals separately. This phase will follow a human-centred design thinking process through workshops to define, ideate, prototype and develop maps, workflows, visual abstracts among other diagrams representing the innovation and its implementation strategies [30,31], focusing on determinants described after specific aim 1 execution [32].

Stakeholders feedback: Local investigators will ask for feedback to regional stakeholders about the Innovation designed in the previous phase. Feedback collection will follow a systematic approach [33]: we will execute focus groups with stakeholders to gather data regarding positives, areas of improvement and negatives of the Innovation. To better contextualise the feedback, focus groups will also gather information concerning the following constructs of the Inner Setting domain in relation to the Innovation: Compatibility, Relative priority, Incentive systems, Mission alignment, and Access to knowledge and information. Focus groups will be recorded, transcribed, analyzed thematically, and presented in codebooks and quotes to inform the following stage.

Innovation refinement and presentation: Local investigators will improve the Innovation according to the feedback from the previous stage. A second version of the Innovation will be presented to stakeholders, including at least the following deliverables [34,35]: Name, Rationale, Goal, Materials, Procedures, Responsibles, Delivery, Location, Dosage and Expected costs. Local investigators will develop causal pathway diagrams for each implementation strategy considered during the Innovation co-design [36].

#### Specific aim 3

The design of the third phase is convergent mixed methods with equal priority: Quan + Qual (Graphic 3) [26]. Local investigators will demonstrate one-on-one to healthcare workers and blood donors a video describing the prototypes, workflows and visual abstracts of the innovation and its implementation strategies. After explaining how the innovation works, we will examine its feasibility, appropriateness and acceptability through a validated survey and in-depth semi-structured interviews [37,38]. We will measure feasibility and appropriateness in healthcare workers involved in the attention of blood donors. We will measure acceptability in a sample of blood donors.

**Figure:**
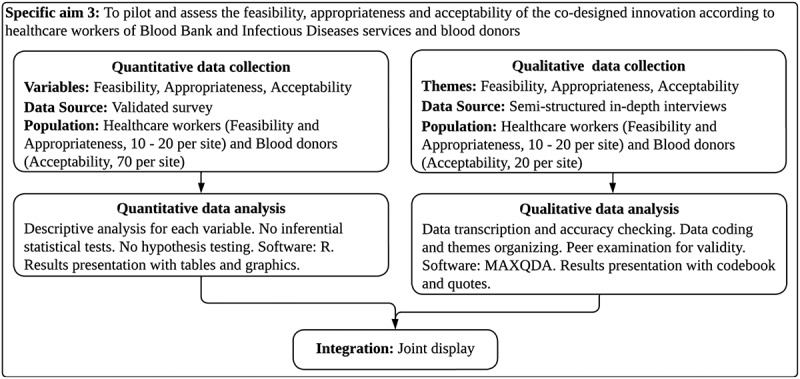
Graphic 3: Convergent mixed methods design for specific aim 3.

### Implementation science framework

The practical problem we are addressing is the failure of the healthcare system in Peru to systematically deliver healthcare to blood donors who may be affected by transfusion-transmissible infections. As a result, these individuals do not receive proper diagnosis confirmation or necessary management. In this study, we will co-design an innovation based on contextual determinants that could function as barriers or facilitators for its implementation.

To accomplish this, we are using the Consolidated Framework for Implementation Research [39]: we will focus on studying the Inner Setting Domain of both ‘Santa Rosa’ and ‘Amazónico’ hospitals as contextual determinants using comprehensive methods, addressing deliverers, recipients and stakeholders. We will also study the Care Continuum of blood donors to better identify areas of improvement through process mapping, a tool from the Systems Engineering approach [40,41].

As part of the situation analysis, two local investigators per site were consulted about their perspectives of the practical problem. The research team deliberated on which constructs from the Inner Setting Domain should be studied in this phase, when and how. The team decided to focus on the study of the following constructs of the Inner Setting domains in the first aims of the project:

During specific aim 1, previous to designing the Innovation, by mixed methods: Structural Characteristics; Available Resources; Relational Connections; Communications; Culture; Tension for Change.

During specific aim 2, while co-designing the Innovation, by qualitative methods: Compatibility; Relative Priority; Incentive Systems; Mission Alignment; Access to Knowledge & Information.

After co-designing an Innovation based on the specific contextual determinants from the Inner Setting and bottlenecks in the Care Continuum, we will evaluate the following Antecedent Assessments as Implementation Determinants during specific aim 3: Feasibility, Appropriateness and Acceptability [22]. We expect this approach will enhance the probability of successful implementation in future phases.

### Study setting

This project will be executed in the Peruvian regions of Madre de Dios and Ucayali. In Madre de Dios, the research site will be the ‘Santa Rosa’ Hospital from the Ministry of Health. In Ucayali, the research site will be the ‘Amazónico’ Hospital from the Ministry of Health. Blood Bank and Infectious Diseases services are mainly involved in the healthcare of blood donor candidates in both sites. Resources and processes between hospitals differ markedly.

### Population

To obtain valid, representative and pragmatic results, we are enrolling participants from three groups: blood donors, healthcare workers and stakeholders. Table 1 defines these populations and their respective eligibility criteria. For in-depth interviews with blood donors, we will balance sex 50/50 to explore potential sex-based differences in risk counseling, results notification, diagnosis, and management within their care continuum. We are not excluding donors who sell their blood, as they also demonstrate a higher prevalence of transfusion-transmissible infections, therefore their perspectives are essential to holistically identify areas for improvement in blood donor care.

**Table 1.** Population and eligibility criteria.

Population	Definition	Inclusion Criteria	Exclusion Criteria
Blood donors	Individuals donating blood either voluntarily or by replacement at “Santa Rosa” and “Amazónico” hospitals. Replacement blood donors are typically relatives or friends of patients who have already used blood units. Although forbidden by Peruvian law, some replacement donors receive payment.	- Age ≥18 years. - Donate blood at either site. - Provide written informed consent.	- Unable to communicate in Spanish. - Withdraws consent at any point.
Healthcare workers	Professionals working within Blood Bank and Infectious Diseases services at “Santa Rosa” and “Amazónico” hospitals: laboratory technicians, nurses, midwives, physicians, administrative staff.	- Work in Blood Bank or Infectious Diseases services at either hospital. - Provide written informed consent.	
Stakeholders	Individuals with influence or interest in the implementation of the Innovation, as identified by local investigators.	- Age ≥18 years. - Identified through stakeholder analysis. - Provide written informed consent.	

### Sampling and sample size

Non-probabilistic, purposive sampling for the three populations.

#### Specific aim 1

##### Blood donors

Health service records: In ‘Santa Rosa’ Hospital in Madre de Dios, we will identify all blood donors from January 2022 to December 2023 who had at least one reactive or indeterminate screening result and follow their medical records for 12 months. We expect at least 200 blood donors with reactive screening results in that period. In ‘Amazónico’ Hospital in Ucayali, we will identify all blood donors from January to December 2023 who had at least one reactive or indeterminate screening result and follow their medical records for 12 months. We expect at least 250 blood donors with reactive screening results in that period.

Interview: We will enroll at least 20 blood donors per site for interviews, evaluate for saturation and increase the sample if needed [42]. Saturation is considered as the stage where no new themes arise from interviews. We will evaluate for thematic saturation to define a proper final sample size of blood donors. The goal of evaluating saturation in this population is to gather sufficient information related to the Care Continuum, according to blood donors’ perspective.

##### Healthcare workers

We will enroll approximately 10–20 individuals per research site, depending on the total number of healthcare workers at Blood Bank and Infectious Diseases services. After finishing interviews with all healthcare workers who participate, we will evaluate collected data for thematic saturation on each site [42]. The goal of evaluating saturation in this population is to explore if the final sample size was sufficient to gather comprehensive information related to the contextual determinants Inner Setting and the Care Continuum, according to healthcare workers’ perspective.

#### Specific aim 2

##### Stakeholders

We will organise at least four focus groups with 5–10 stakeholders per site, evaluate for saturation and increase the number of focus groups if needed to ensure thematic saturation [42]. We will invite at least two stakeholders per profile per site among the following: Healthcare provider from Blood Bank service, Healthcare provider from Infectious Diseases service, Frequent blood donor, First-time blood donor, Community leader, Hospital administrator, Hospital software developer, Government representative, Non-Government Organization representative, among others identified during specific aim 2. The goal of evaluating saturation in this population is to gather sufficient feedback about the Innovation, according to stakeholders’ perspective.

#### Specific aim 3

##### Blood donors

Survey: We will enroll 70 blood donors per site to respond to the Acceptability questions from the survey developed by Weiner et al. (2017) [37]. The survey is measured in a 5-Point Likert Scale. To calculate the sample size, we have considered a desired confidence interval width of 0.75, a standard deviation = 1.5 meaning moderate variability of responses, and a confidence interval of 95%. Given those measurements, the initial sample size calculation was 62 [43], which we inflated to 70 to account for potential losses.

Interview: We will enroll at least 20 blood donors per site for interviews, evaluate for saturation and increase the sample if needed. The sample for interviews will be collected from those who responded to the previous survey. We will evaluate for thematic saturation to define a proper final sample size [42]. The goal of evaluating saturation in this population is to gather sufficient information related to the implementation determinant Acceptability of the Innovation, according to blood donors’ perspective.

##### Healthcare workers

Survey and Interview: We will enroll approximately 10–20 individuals per research site, depending on the total number of healthcare workers at Blood Bank and Infectious Diseases services. After finishing surveys and interviews with all healthcare workers who participate, we will evaluate the collected data for thematic saturation on each site [42]. The goal of evaluating saturation in this population is to explore if the final sample size was sufficient to gather comprehensive information related to the implementation determinants Feasibility and Appropriateness of the Innovation, according to healthcare workers’ perspective.

### Recruitment

#### Specific aim 1

##### Blood donors

Health service records: Identification of previous blood donors with a reactive or indeterminate screening result will be done from record books and serum storages. Medical records for follow up will be asked to the file of the Admission service of the Statistics and Informatics unit, as well as searched in the Infectious Diseases service records.

Interview: Local investigators will briefly explain in groups the study and the purpose of interviews with visual support. After group explanation, researchers will ask blood donors to participate in the project. This process will be held previous to beginning the blood donation process. If blood donors accept, they will be asked for consent and enrolled before the blood donation process starts and interviewed afterwards the same day.

##### Healthcare workers

Local investigators and service heads will explain the project to healthcare workers in different schedules during a week before shift starts. After the first group explanation, each member of the healthcare service will be addressed separately to further explain the project and proceed with the informed consent in case they accept to participate. After signing the informed consent, specific dates for interviews will be scheduled. Absent healthcare workers will be addressed as soon as they return to work. The informed consent for healthcare workers will also include the assessments to be done during specific aim 3.

#### Specific aim 2

##### Stakeholders

We will contact stakeholders through two methods: direct contact and secondary invitation (‘snowball sampling’). After stakeholder analysis, direct contact will be through email, letter and in-person meeting as needed. Every firstly identified stakeholder will be asked to invite other two persons who they believe are also stakeholders. Stakeholders will be invited to participate in the following stages of the specific aim 2 and asked to consent.

#### Specific aim 3

##### Blood donors

Local investigators will ask blood donors to participate after briefly explaining the study and the purpose of the survey and interviews previous to entering the blood bank for beginning the blood donation process. If blood donors accept, they will be enrolled before the blood donation process starts, and surveyed and interviewed afterwards. Blood donors will have the option to participate only in the survey or both the survey and interview.

##### Healthcare workers

New healthcare workers will be addressed separately to further explain the project and proceed with the informed consent in case they accept to participate. Healthcare workers will be addressed individually to schedule specific dates for surveys and interviews. Absent healthcare workers will be addressed as soon as they return to work.

### Outcomes

Outcomes will be evaluated from different population’s perspectives and methods, as summarised in Table 2. We will explore as primary outcomes both Contextual determinants and Implementation determinants. Contextual determinants (Inner Setting and Care Continuum, described in Table 3) will influence the Innovation design. Implementation determinants (Feasibility, Appropriateness and Acceptability, described in Table 4) will likely predict implementation success. Additionally, we will collect demographic information from blood donors (Table 5).

**Table 2.** Outcomes, methods for assessment and respective population.

Primary outcome		Quantitative component	Qualitative component
Contextual determinants	Inner Setting	Secondary data Structural Characteristics Available Resources	Interviews to healthcare workers Relational Connections Communications Culture Tension for Change Focus groups with stakeholders Compatibility Relative Priority Incentive Systems Mission Alignment Access to Knowledge & Information
	Care Continuum	Secondary data Risk counseling Results notification Diagnosis Management	Non-participant observation; Interviews to blood donors; Interviews to healthcare workers Risk counseling Results notification Diagnosis Management
Implementation determinants	Feasibility	Survey to healthcare workers	Interviews to healthcare workers
	Appropriateness	Survey to healthcare workers	Interviews to healthcare workers
	Acceptability	Survey to blood donors	Interviews to blood donors

**Table 3.** Contextual determinant outcomes.

Outcome	Component	Definition	Operationalization	Time point
Inner Setting [39]	Structural Characteristics	Infrastructure components supporting functional performance of both Blood Bank and Infectious Diseases services	Physical Infrastructure: Layout and configuration of space, physical space availability; Categorical nominal Information Technology Infrastructure: Technological systems for tele-communication, electronic documentation, and data storage, management, reporting, and analysis; Categorical nominal Work Infrastructure: Organization of tasks and responsibilities within and between individuals and teams, and general staffing levels; Categorical nominal	Before Innovation design, during the execution of specific aim 1
	Available Resources	Resources supporting functional performance of both Blood Bank and Infectious Diseases services	Funding sources and availability; Categorical nominal Materials and equipment availability; Categorical nominal	
	Relational Connections	Formal and informal relationships, networks, and teams within and across both Blood Bank and Infectious Diseases services	Emerging themes on quality of relationships and networks	
	Communications	Formal and informal information sharing practices within and across both Blood Bank and Infectious Diseases services	Emerging themes on quality of information sharing practices	
	Culture	Values, beliefs, and norms across both Blood Bank and Infectious Diseases services	Emerging themes around caring, supporting, and addressing the needs and welfare of blood donors and healthcare workers	
	Tension for Change	The degree to which healthcare delivery to blood donors is intolerable and needs to change	Emerging themes on intolerability of current situation	
	Compatibility	The degree to which the innovation fits with workflows, systems, and processes of both Blood Bank and Infectious Diseases services	Emerging themes on innovation fitting workflows, systems, and processes	During Innovation design, during the execution of specific aim 2
	Relative Priority	The degree to which implementing the innovation is important compared to other initiatives	Emerging themes on the importance of implementing the innovation	
	Incentive Systems	The degree to which incentives, rewards, disincentives or punishments support implementation of the innovation	Emerging themes on incentives, rewards, disincentives or punishments	
	Mission Alignment	The degree to which implementing the innovation is in line with the overarching goals of both Blood Bank and Infectious Diseases services	Emerging themes on the innovation aligning the inner setting mission	
	Access to Knowledge & Information	The degree to which guidance and training is accessible to implement innovation	Emerging themes on accessibility to guidance and training in the innovation	
Care Continuum	Risk counseling	The process of addressing risk factors for having a transfusion-transmissible infection that prevent blood donors to donate	Effective risk counseling, Categorical binomial (yes, no) Service of risk counseling, Categorical nominal Emerging themes on the effectiveness and ways to improve risk counseling	Before Innovation design, during the execution of specific aim 1
	Results notification	The process of informing blood donors about their reactive or indeterminate results, and the need to further evaluation	Effective notification, Categorical binomial (yes, no) Notification method, Categorical nominal (“Phone-call”, “Letter”, “Text message/Short Message Service (SMS)”, “WhatsApp”, “Other”) Time to event since screening up to 12 months, Quantitative discrete Emerging themes on the processes, effectiveness and ways to improve results notification	
	Diagnosis	The process of confirming or ruling out a transfusion-transmissible infection after obtaining a reactive or indeterminate screening result	Diagnosis effective evaluation (confirmation or rule out), Categorical binomial (yes, no) Hospital department responsible for diagnosis, Categorical nominal (“Infectious Diseases”, “Midwifery”, “Internal or General Medicine”, “Nursery”, “Other”) Time to event since screening up to 12 months Emerging themes on the processes, effectiveness and ways to improve diagnosis	
	Management	The process of engaging the blood donor in tertiary prevention strategies, consisting in specific pharmacological therapies, counseling or follow-up evaluations	Effective management, Categorical binomial (yes, no) Adequate management, Categorical binomial (yes, no) Hospital service where management was done, Categorical nominal (“Infectious Diseases”, “Midwifery”, “Internal or General Medicine”, “Nursery”, “Other”) Time to event since screening up to 12 months, Quantitative discrete Emerging themes on the processes, effectiveness and ways to improve management

**Table 4.** Implementation determinant outcomes.

Outcome	Definition	Operationalization	Time point
Feasibility	The extent to which the innovation can be successfully carried out within the hospital, according to the healthcare workers of Blood Bank and Infectious Diseases services [22]	Four survey questions per outcome [37] 5-Point Likert Scale: 1, Completely disagree 2, Disagree 3, Neither agree nor disagree 4, Agree 5, Completely agree Interview questions Emerging themes on feasibility, appropriateness and acceptability of the innovation	After Innovation design, during the execution of specific aim 3
Appropriateness	The perceived relevance and compatibility of the innovation to address the practical problem within the hospital, according to the healthcare workers of Blood Bank and Infectious Diseases services [22]		
Acceptability	The extent to which the innovation is perceived as agreeable, according to blood donors [22]

**Table 5.** Demographic data from blood donors.

Variable	Definition	Operationalization	Time point
Name	Full name of blood donor	Categorical nominal, Identifier	Before and After Innovation design, during the execution of specific aim 1 and 3
Identification	Numerical identifier from the national identity card	Categorical nominal, Identifier	
Age	Time in years between date of enrollment and date of birth	Quantitative discrete	
Sex	Biological reproductive characteristic	Categorical binomial (Male, Female)	
Date first assessment	First visit to blood bank	Quantitative discrete	
Reason for donation	Reason for which the blood donors approaches to the blood bank	Categorical binominal (Voluntary, Replacement)	
Type of donation	Type of donation in terms of frequency	Categorical binomial (First time donor, Recurrent donor)	

### Data collection

We will use Research Electronic Data Capture (REDCap) [44] to collect primary (surveys, interviews, focus groups and non-participant observation) and secondary data (health service records). REDCap is a secure, web-based application that supports data encryption, audit trails, and role-based access controls to ensure data confidentiality and integrity. Data will be coded during collection and anonymized prior to export for analysis. Identifiable data will be accessible only to the principal investigator (PI) for verification, quality control, and auditing. All data will be stored in a password-protected REDCap project, and weekly backups will be saved to the PI’s encrypted personal computer. Data from both study sites will be reviewed weekly for accuracy, consistency, and completeness by the PI.

#### Specific aim 1

We will use a data collection form for gathering information regarding the care continuum of blood donors from hospital services records and archives. We will use a guide for non-participatory observation for further studying the processes involved in the care continuum of blood donors. We will also use in-depth semi-structured interview guides for gathering blood donors’ and healthcare workers’ perspectives on contextual determinants.

#### Specific aim 2

We will use a focus group guide for gathering feedback from stakeholders on the Innovation design.

#### Specific aim 3

We will use translated validated surveys [37,38] for gathering information on Feasibility (4 questions) and Appropriateness (4 questions) according to healthcare workers, and Acceptability (4 questions) according to blood donors. We will validate these tools through forward-backward translation, participatory co-design, and piloting with local researchers and stakeholders during specific aim 2. Adjusted tools will be used after IRB approval. We will also use in-depth semi-structured interview guides for gathering information on Feasibility and Appropriateness according to healthcare workers, and Acceptability according to blood donors.

### Data management

Codification: Prospective participants will have a unique alpha-numerical code since enrollment by which we will aggregate responses. Codification will gather mixed information from the Site, Role of participant, Date and Count separated by a ‘_’.

Site: MDD (Madre de Dios), UCA (Ucayali)

Role of participant: BD (Blood donor), HW (healthcare worker at Blood Bank or Infectious Diseases), ST (stakeholder)

Date: Year_month_day

Count: Collection count per day of specific population by site: 001, 002, 003 …

Example: MDD_BD_25_02_20_002: Participant from Madre de Dios, blood donor enrolled on February 20th of 2025, participant number 02 of the day

Transcription: Interviews and focus groups will be recorded and transcribed verbatim. In case the participant does not desire to be recorded, annotations will be made.

Storage: Identifiable sensitive data from participants will be deleted from databases. Codified and anonymised data will be stored physically for 10 years at the PARACAS office at the Instituto de Medicina Tropical ‘Alexander von Humboldt’ and digitally in REDCap hosted in Universidad Peruana Cayetano Heredia if participants consent to do so.

### Data analysis

Table 6 summarises the analysis processes and deliverables of the mixed-methods approach.

**Table 6.** Data analysis processes.

Analysis type	Methods	Deliverables
Quantitative Component: Secondary Data & Surveys	Quantitative variables: Median, interquartile range (IQR) Categorical variables: Absolute (N) and relative (%) frequencies	Descriptive statistics, no hypothesis testing Tables and figures summarizing distributions
Qualitative Component: Interviews, Focus Groups & Non-Participant Observations	Initial coding: Deductive coding to organise expected responses based on a preliminary set of codes Coding refinement: Modification of initial codes based on emerging themes; iterative review of transcripts Developing themes: Selection of the most important themes arising from the codes Thematic analysis: Identification of relationships between themes; use of thematic maps Triangulation: Comparison of themes across blood donors and healthcare workers to validate findings	Coding Matrix with revised codes, themes and definitions Thematic Maps illustrating linkages between key themes and subthemes Narrative synthesis of themes and their practical interpretation
Integration	Synthesis and contextual interpretation of Inner Setting through a narrative discussion Synthesised presentation of findings through joint display of Care Continuum and Implementation Determinants (Feasibility, Appropriateness, Acceptability).	Narrative discussion of Inner Setting Joint Displays of Care Continuum and Implementation Determinants

## Discussion

In Peru and other low- and middle-income countries, where replacement blood donations predominate, systematic transfusion-transmissible infections screening create opportunities for infection control in addition to ensuring blood safety. This study will develop a feasible, appropriate and acceptable innovation to improve the processes of screening results notification, diagnosis and management for blood donors potentially affected by transfusion-transmissible infections, such as HIV and HTLV-1/2.

CFIR defines implementation across five domains: innovation, outer setting, inner setting, individuals, and implementation process [23]. A recent revision introduced antecedent assessments (feasibility, appropriateness, and acceptability) as key determinants of adoption and sustainability [39]. This study will evaluate antecedent assessments using a convergent mixed methods approach, combining Weiner et al. (2017) validated survey [37] with semi-structured interviews [39]. Additionally, process mapping from the Systems Engineering approach will identify inefficiencies in blood donor healthcare pathways, informing targeted interventions [40,41].

After an in-depth analysis of the Care Continuum of blood donors and the Inner Settings of the Blood Bank and Infectious Diseases services in two hospitals from the Peruvian Amazon, we will co-design an innovation to address critical gaps, employing the hospital information system software. To increase the likelihood of future successful implementation, this study design also includes stakeholder engagement strategies since the early development of the innovation. The resulting innovation has the potential for broader adaptation across Peru and could inform national blood donation policy reforms. We recommend researchers to adapt the approach presented in this protocol to inform implementation research in their own settings.

## Conclusion

This study will generate a feasible, appropriate, and acceptable innovation to improve transfusion-transmissible infections diagnosis and management among replacement blood donors. Participant enrollment, data collection and other project activities have not been implemented at submission. Findings will inform scalable, context-specific interventions to strengthen blood donor care and guide policy.
